# Massivste epigastrische Schmerzen bei einem 59-jährigen Patienten

**DOI:** 10.1007/s00108-020-00905-x

**Published:** 2020-11-19

**Authors:** S. Welland, C. Janssen, K. I. Ringe, G. Höglinger, M. P. Manns, Ingmar Mederacke

**Affiliations:** 1 KIinik für Gastroenterologie, Hepatologie und Endokrinologie, Medizinische Hochschule Hannover, Carl-Neuberg-Str. 1, 30625 Hannover, Deutschland; 2 Klinik für Neurologie, Medizinische Hochschule Hannover, Hannover, Deutschland; 3 Institut für Diagnostische und Interventionelle Radiologie, Medizinische Hochschule Hannover, Hannover, Deutschland

**Keywords:** Akute Neuroborreliose, Radikulitis, Polyneuropathien, Erythema chronicum migrans, Liquordiagnostik, Lyme neuroborreliosis, acute, Radiculitis, Polyneuropathies, Erythema chronicum migrans, Diagnostics, cerebrospinal fluid

## Abstract

Abdominelle Schmerzen sind oft Konsultationsanlass in Arztpraxen und Notaufnahmen. Die häufigsten Differenzialdiagnosen lassen sich mit gut verfügbarer, kosteneffektiver und risikoarmer Diagnostik (Laboruntersuchungen, Sonographie, Gastroskopie) bestätigen. Zum Ausschluss seltener Ursachen, wie kleiner solider oder hämatologischer Malignome, Stoffwechselstörungen oder Polyneuropathien unterschiedlichster Genese, kann eine erweiterte Diagnostik erforderlich sein. Im Folgenden stellen wir den Fall eines Patienten mit massivsten epigastrischen Beschwerden infolge einer Neuroborreliose vor und rekapitulieren die diagnostischen Schritte zur Abklärung des abdominellen Schmerzes.

## Abstract (en)

Abdominal pain is a frequent cause of consultation to doctors’ offices and emergency rooms. The most common differential diagnoses can be confirmed with readily available, cost-effective, and low-risk diagnostic tools such as laboratory tests, ultrasound, or gastroscopy. Additional diagnostic tests are required to exclude rare causes such as small, solid, or hematological malignancies, metabolic disorders, or polyneuropathies of varying origin. In the following, we present the case of a patient with severe epigastric pain due to neuroborreliosis, and recapitulate the diagnostic steps for clarifying abdominal pain using this example.

## Anamnese

Die Erstvorstellung des 59-jährigen Patienten erfolgte über die zentrale Notaufnahme unserer Klinik in Begleitung seiner Ehefrau. Er hatte stärkste, seit einigen Tagen bestehende Oberbauchschmerzen. Am Vortag war der Patient von einer Urlaubsreise aus Österreich zurückgekehrt. Bei dort begonnener Symptomatik war eine Gastroskopie ohne wegweisenden Befund geblieben. Bei bereits länger bestehendem Halswirbelsäulen(HWS)-Syndrom wurde eine Therapie mit dem nichtsteroidalen Antirheumatikum Ibuprofen unter Säureblockade (Pantoprazol) eingeleitet.

Bei Aufnahme in domo wurden die Schmerzen als massiv und brennend beschrieben mit vermehrtem Auftreten in der Nacht sowie Ausstrahlung in den Rücken. Gewichtsverlust und Nachtschweiß wurden verneint.

An Vorerkrankungen bestanden neben dem HWS-Syndrom ein arterieller Hypertonus unter einer Kombinationstherapie mit Nebivolol und Olmesartan sowie eine zurückliegende depressive Episode. Operative abdominelle Eingriffe waren bislang nicht erfolgt.

## Klinischer Befund

Bei Aufnahme zeigte sich der 59-jährige Patient in reduziertem Allgemeinzustand bei normalem Ernährungszustand (82 kg bei 188 cm, Body-Mass-Index 23,2 kg/m^2^). Die Vitalparameter waren normwertig und es bestand kein Fieber. In der körperlichen Untersuchung zeigte sich ein druckschmerzhaftes Epigastrium ohne Zeichen eines Peritonismus. Stuhlgang und Miktion waren unauffällig.

## Diagnostik

### Labordiagnostik

Im *Aufnahmelabor* inklusive *Urinuntersuchung* (δ-Aminolävulinsäure und Porphobilinogen) zeigte sich abgesehen von einer milden normozytär-normochromen Anämie sowie einer isolierten, geringgradigen Erhöhung der Aspartat-Aminotransferase ein unauffälliger Befund. *Serologisch* wurden eine akute Infektion mit Herpesviren (Herpes-simplex-Virus, Varizella-Zoster-Virus, Zytomegalievirus [CMV], Epstein-Barr-Virus [EBV]) oder Hepatitisviren sowie eine Borrelieninfektion bei anamnestisch bestehendem Zeckenbiss ausgeschlossen.

### Sonographie

Sonographisch ergaben sich bei bisher symptomloser Cholezystolithiasis lediglich eine leichte Prostatahypertrophie sowie minimale Mengen freier Flüssigkeit (Recessus hepatorenalis und splenorenalis).

### Endoskopie

Eine erneute *Gastroskopie* erbrachte ein in Abheilung befindliches Ulkus im Bereich der Z‑Linie sowie eine leichtgradige Refluxösophagitis (Los-Angeles-Stadium A) ohne weitere pathologische Befunde.

### Kardiale Diagnostik

Bei unauffälligem *Elektrokardiogramm* konnte ein Myokardinfarkt *laborchemisch* ausgeschlossen werden. *Echokardiographisch* zeigte sich eine gute Pumpfunktion ohne Klappenvitien. *Computertomographie(CT)-morphologisch* ergaben sich keine Hinweise auf eine Lungenarterienembolie oder eine Aortendissektion.

### Magnetresonanztomographie

**Figure 1:**
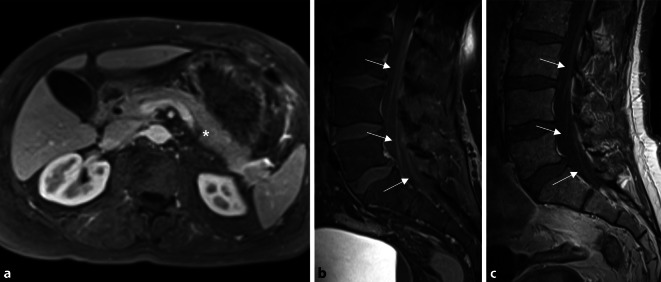

Bei opiatrefraktären Schmerzen wurde *Magnetresonanz(MR)-morphologisch* ein die Perineuralscheide infiltrierendes Pankreaskarzinom ausgeschlossen (Abb. 1). Bei zwischenzeitlich mitbestehenden Schmerzen in beiden proximalen Oberschenkeln ergab eine *Magnetresonanztomographie der Wirbelsäule* eine deutliche Kontrastmittelanreicherung der lumbalen Nervenwurzeln im Sinne einer Radikulitis (Abb. 1).

### Liquordiagnostik

**Figure 2:**
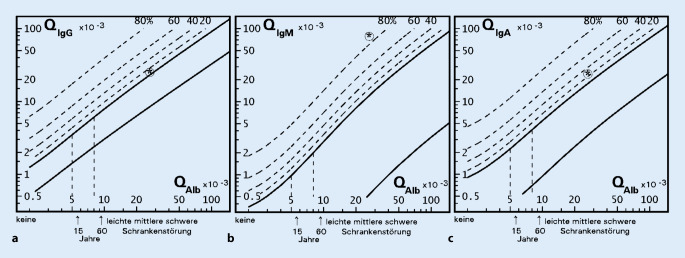

Die Untersuchung des Liquors zeigte ein entzündliches Zellbild mit lymphomonozytärer Pleozytose, schwerer Schrankenstörung und intrathekaler Immunglobulinsynthese mit oligoklonalen Banden von Typ 2 (Tab. 1; Abb. 2), vereinbar mit einem infektiösen Geschehen passend zu einer Neuroborreliose. Die Diagnose konnte schließlich durch den Nachweis von borrelienspezifischen Antikörpern im Liquor bei persistierend negativer *serologischer Untersuchung* im Blut bestätigt werden (Tab. 2). Eine nachträglich durchgeführte Bestimmung von Borrelienantikörpern im Serum mittels *Immunblot* ergab zum Zeitpunkt der Diagnosestellung einen positiven Befund für eine singuläre Bande (Immunglobulin G gegen „variable major protein-like sequence, expressed“ [VlsE]).

**Table 1:** 

	Zeitpunkt Diagnose	3 Wochen nach Therapiestart	Einheit	Referenzwert
*Zytologie*				
Zellzahl Liquor	70	31,3	Zellzahl/µl	<5,0
Erythrozyten	1,0	0,3	Zellzahl/µl	<1,0
Lymphozyten	89	98	%	–
Monozyten	10	5	%	–
Granulozyten	0	0	%	–
Plasmazellen	1	0	%	–
*Proteine*				
Gesamtprotein	1713	598	mg/l	–
Liquor-Alb	1060	460	mg/l	–
Liquor-IgG	206	54,6	mg/l	–
Liquor-IgA	51,9	12,4	mg/l	–
Liquor-IgM	51,1	6,8	mg/l	–
Serum-Alb	39,9	42,3	g/l	–
Serum-IgG	8,21	8,8	g/l	–
Serum-IgA	2,18	2,23	g/l	–
Serum-IgM	0,65	0,86	g/l	–
Q‑Alb	26,57	10,87	–	–
Q‑IgG	25,09	6,19	–	–
Q‑IgA	23,81	5,56	–	–
Q‑IgM	78,50	7,99	–	–
IgG-Index	0,944	0,569	–	–
Lokale Synthese IgA	25,7	0,0	%	–
Lokale Synthese IgG	7,9	0,0	%	–
Lokale Synthese IgM	84,5	59,5	%	–
*Oligoklonale Banden*	Typ 2	Typ 3	–	–

*Alb* Albumin, *IgA* Immunglobulin A, *IgG* Immunglobulin G, *IgM* Immunglobulin M, *Q* Quotient

**Table 2:** 

	Zeitpunkt Diagnose	Einheit	Bewertung	Interpretationsbereich
*Serum*				
*Borrelia-burgdorferi*-Komplex-IgG (ELISA)	13,49	U/ml	Negativ	<20 (grenzwertig: 20–24)
*Borrelia-burgdorferi*-Komplex-IgM (ELISA)	16,97	U/ml	Negativ	<20 (grenzwertig: 20–24)
*Liquor*				
*Borrelia-burgdorferi*-Komplex-IgG (ELISA)	–	–	Positiv	Negativ
*Borrelia-burgdorferi*-Komplex-IgM (ELISA)	–	–	Positiv	Negativ
*Borrelia*-Liquor/Serum-Antikörperindex für IgG	15,18	–	Positiv	≤1,3 (grenzwertig: 1,3–1,5)
*Borrelia*-Liquor/Serum-Antikörperindex für IgM	15,92	–	Positiv	≤1,3 (grenzwertig: 1,3–1,5)

*ELISA* „Enzyme-linked immunosorbent assay“, *IgG* Immunglobulin G, *IgM* Immunglobulin M

## Diagnose

- Neuroborreliose nach Zeckenbiss mit diffuser Schmerzsymptomatik

## Therapie und Verlauf

Bei entzündlichem Liquorbefund wurde eine empirische antiinfektive Therapie mit Ceftriaxon und Aciclovir begonnen. Unter Erwägung einer autoimmunologischen Ursache der nachgewiesenen axonalen Polyneuropathie wurde ein Steroidstoß mit Methylprednisolon ergänzt. Mit Erhalt des Nachweises von Borrelienantikörpern im Liquor wurde die antiinfektive Therapie mit Ceftriaxon über insgesamt 14 Tage intravenös durchgeführt. Unter einer Medikation mit Pregabalin war der Patient erstmalig symptomfrei. Eine Woche nach Beendigung der antiinfektiven Therapie erfolgte eine erneute stationäre Aufnahme bei neu aufgetretener Hypästhesie und Kribbelparästhesien im linken Bein (Dermatome L5–S1 links). Bei rückläufiger, aber weiterhin erhöhter Liquorzellzahl (Tab. 1) sowie MR-morphologischer Kontrastmittelanreicherung in der Cauda equina konnte nach erneuter 14-tägiger Therapie mit Ceftriaxon eine anhaltende Remission erreicht werden.

## Diskussion

### Differenzialdiagnosen bei abdominalen Schmerzen

Oftmals führen bei epigastrischen Schmerzen eine gründliche Anamnese und die klinische Untersuchung schon zu einer Verdachtsdiagnose, die durch gut verfügbare, wenig invasive und kosteneffektive apparative Maßnahmen bestätigt oder ausgeschlossen werden kann. So zählt zur Abklärung neben der laborchemischen Basisdiagnostik eine Sonographie des Abdomens, die Aufschluss über strukturelle Veränderungen an den Bauchorganen (z. B. Raumforderungen) oder Hinweise auf Gefäßverschlüsse geben kann. Die Aussagekraft einer Sonographie kann jedoch durch Faktoren wie das Körpergewicht, Immobilität des Patienten, Meteorismus oder fehlende Routine des Untersuchers limitiert sein. Zum Ausschluss eines Aortenaneurysmas, einer Dissektion oder einer mesenterialen Ischämie ist daher oftmals eine CT notwendig. Im weiteren Vorgehen gehört auch eine Gastroskopie zur Abklärung epigastrischer Beschwerden.

Nach Ausschluss häufiger Ursachen abdomineller Schmerzen müssen auch seltenere Ursachen, wie eine Porphyrie, in Betracht gezogen werden. Auch hämatologische Grunderkrankungen (Leukämien, hämolytische Anämien wie Sichelzellanämie oder Sphärozytose) sollten durch Anfertigung eines Differenzialblutbilds mit Blutausstrich zur mikroskopischen Beurteilung ausgeschlossen werden. Selten kann ein Pankreaskarzinom im frühen Stadium durch eine Perineuralscheideninfiltration zu ausgeprägten Oberbauchbeschwerden führen; eine solche Infiltration ist am besten MR-morphologisch zu erfassen.

Auch metabolische Störungen wie die Hyperkalzämie, eine Pseudoperitonitis diabetica im Rahmen eines Diabetes mellitus, eine thyreotoxische Krise oder Addison-Krise sowie eine Urämie sollten ausgeschlossen werden, wobei die Laboruntersuchung hier wegweisend ist. Hinweise auf eine Bleivergiftung als sehr seltene Ursache, die neben Bauchschmerzen auch zu auffälliger Färbung der Gingiva, Einschränkungen der Hämatopoese und Radialislähmungen führen kann, geben Erythrozyten mit basophiler Tüpfelung.

Auch Neuropathien können ursächlich für anders nicht zu erklärende abdominelle Schmerzen sein

Zuletzt können auch Neuropathien ursächlich für anders nicht zu erklärende abdominelle Schmerzen sein. Im Falle von dermatombezogenen Schmerzen muss auch bei fehlender typischer Effloreszenz ein Herpes zoster serologisch ausgeschlossen werden [1]. Auch infektiologische Ursachen wie eine Infektion mit „human immunodeficiency virus“, CMV oder EBV sowie Hepatitis B, C oder E, aber auch bakterielle Erreger wie Mykoplasmen, Borrelien oder das *Clostridioides-difficile*-Toxin können eine akut verlaufende Neuropathie hervorrufen [2, 3]. Auch die Neuroborreliose, ausgelöst durch eine Polyradikulitis spinaler Nerven, stellt eine mögliche seltene Ursache für abdominelle Schmerzen dar.

### Epidemiologie der Neuroborreliose

Häufig kann eine genaue Anamnese bei einem Zeckenbiss Hinweise auf die Wahrscheinlichkeit einer Borrelieninfektion liefern. Bereits Daten aus den 1990er-Jahren belegen eine deutlich höhere Inzidenz der Lyme-Borreliose in Österreich mit etwa 120 Erkrankten pro 100.000 Einwohner im Vergleich zu Deutschland mit 26–41 Fällen pro 100.000 Einwohner [4]. Da der Holzbock als übertragende Zecke ab einer Außentemperatur von 6 °C aktiv ist, erfolgt eine Übertragung in den Monaten von Februar/März bis Oktober/November mit einem Häufigkeitsgipfel der Neuroborreliose in den Monaten Juli und August [4, 5].

### Klinische Manifestation

Eine Neuroborreliose tritt bei etwa 10–15 % aller klinisch apparenten Borrelieninfektionen auf. Die häufigste Manifestationsform mit etwa 10 % stellt eine Beteiligung des peripheren Nervensystems mit inflammatorischer Polyradikulitis von Spinalnerven oder Hirnnerven mit segmental-radikulärer, häufig nächtlicher Schmerzsymptomatik in Form des Bannwarth-Syndroms dar, in 2–4 % der Fälle kann es zudem zu einer Beteiligung des zentralen Nervensystems im Sinne einer Meningitis oder seltener einer Myelitis oder Enzephalitis kommen. Begleitend können weitere Symptome wie Kopfschmerz, Fatigue, Parästhesien sowie periphere Nervenlähmungen der Extremitäten oder Hirnnerven (häufig des N. facialis) bestehen [6]. Typischerweise sprechen die Schmerzen schlecht bis gar nicht auf eine herkömmliche analgetische Therapie an und werden wie neuropathische Schmerzen als brennend und bohrend beschrieben [7]. Symptome treten in einem Zeitraum von 2 bis 18 Wochen nach Infektion auf (im Durchschnitt nach 4–6 Wochen; [8]). Nur in einem Drittel der Fälle ist ein Zeckenbiss sowie in 25–50 % ein Erythema chronicum migrans erinnerlich. Bei der Mehrheit, jedoch nicht bei allen Patienten entwickeln sich im Verlauf von ein bis 4 Wochen überwiegend motorisch-neurologische Ausfälle, die hauptsächlich im Bereich der Hirnnerven lokalisiert sind [9].

### Diagnostik

Im Serum können borrelienspezifische Antikörper gemessen werden, die mit einer Latenz von 4 bis 6 Wochen nach Infektion im peripheren Blut nachweisbar sind. Bei Patienten mit Erythema migrans kann in bis zu 50 % der Fälle die serologische Antikörpertestung unauffällig bleiben [10]. Bei begründetem Verdacht auf eine Neuroborreliose sollte daher eine Liquorpunktion durchgeführt werden, in der sich typischerweise eine lymphozytäre Pleozytose mit Erhöhung des Gesamteiweißes im Sinne einer Schrankenstörung ergibt [11]. Die definitive Diagnose der Erkrankung erfolgt durch Nachweis einer intrathekalen Synthese borrelienspezifischer Antikörper.

### Therapie der akuten Neuroborreliose

Da auch Jahre nach einer Borrelieninfektion noch Immunglobulin-M-Antikörper nachweisbar sein können, sollte die Indikation zur Therapie immer im klinischen Kontext gestellt werden. Insgesamt kann die Neuroborreliose durch die Gabe von Doxycyclin, Ceftriaxon, Cefuroxim oder Penicillin G gut therapiert werden. Die orale Doxycyclintherapie ist der parenteralen Therapie mit β‑Laktam-Antibiotika nicht unterlegen.

Leitliniengerecht ist eine antiinfektive Therapie über eine Dauer von 14 Tagen bei früher Neuroborreliose empfohlen, da Studien bei Verlängerung der Therapiedauer keinen Vorteil zeigen konnten [12]. Ein Jahr nach Behandlung sind 90 % der Patienten symptomfrei [13]. Bei chronischer Neuroborreliose sollte eine längere Therapie für 14–21 Tage angestrebt werden [14].

## Fazit für die Praxis

Bei der Abklärung epigastrischer Beschwerden sollten nach Ausschluss häufiger Ursachen auch seltenere Differenzialdiagnosen wie die Neuroborreliose berücksichtigt werden, die insbesondere auch aufgrund der guten Therapierbarkeit nicht übersehen werden sollte. Eine Neuroborreliose kann auch ohne Erythema chronicum migrans auftreten, auch fällt die serologische Untersuchung auf Borrelien im Blut nicht immer positiv aus, sodass bei Verdacht eine Liquorpunktion zur Diagnosestellung erfolgen sollte.
